# Expression of thyroid transcription factor-1 is associated with a basal-like phenotype in breast carcinomas

**DOI:** 10.1186/1746-1596-8-80

**Published:** 2013-05-15

**Authors:** Tor A Klingen, Ying Chen, Pål Suhrke, Ingunn M Stefansson, Marian D Gundersen, Lars A Akslen

**Affiliations:** 1 Department of Pathology, Vestfold Hospital Trust, Tønsberg, Norway; 2 Department of Radiology, Vestfold Hospital Trust, Tønsberg, Norway; 3 Centre for Cancer Biomarkers, The Gade Institute, University of Bergen, Bergen, Norway; 4 Department of Pathology, Haukeland University Hospital, Bergen, Norway

**Keywords:** Thyroid transcription factor-1, Breast cancer, Basal-like phenotype, Adverse prognostic factors

## Abstract

**Background:**

The differential diagnosis between primary and secondary breast cancers might be difficult, especially in poorly differentiated tumors. Thyroid Transcription Factor-1 (TTF-1) has been regarded as a reliable marker for lung or thyroid origin, with only occasional positive staining in other tumors. However, positive cases have recently been reported among primary breast carcinomas.

**Methods and results:**

Here, we analyzed expression of TTF-1 protein (clone SPT24) by immunohistochemical staining of sections from paraffin embedded tumor samples in 247 primary breast cancers from the population-based Norwegian Breast Cancer Screening Program. Positive staining (weak or strong) was observed in 7 cases (2,8%). As novel observations, positivity was demonstrated more frequently in estrogen receptor negative cases (14,0% vs. 1,4%; p = 0,004), highly proliferative tumors (8,8% vs. 1,1%; p = 0,008), tumors with a basal-like phenotype by showing expression of CK5/6 and/or P-cadherin (11,1% vs. 1,4%; p = 0,01), and tumors with blood vessel invasion (9,7% vs. 1,9%; p = 0,04). Also, TTF-1 was associated with histological grade 3 tumors compared with grade 1 or 2 tumors (7,7% vs. 1,5%; p = 0,04) as well as lymph node positive cases (5,2% vs. 1,8%; p = 0,03).

**Conclusions:**

Our population-based findings indicate that TTF-1 may be positive in approximately 3% of primary breast cancers, and positivity indicates an association with adverse prognostic factors.

**Virtual slides:**

The virtual slide(s) for this article can be found here: http://www.diagnosticpathology.diagnomx.eu/vs/8313753509421182

## Introduction

Thyroid Transcription Factor-1(TTF-1), also known as Nkx2.1 or thyroid-specific enhancer-binding protein, regulates genes in the thyroid, lungs, and diencephalon during embryogenesis [1,2]. Thus, TTF-1 has been regarded as a reliable marker for tumors originating in lung or thyroid tissues. TTF-1 is expressed in 72% of adenocarcinomas of the lung [2] and can be a useful marker in identifying lung tissue as a primary origin of metastases, for instance to the breast. However, recent studies have demonstrated that this marker might also be positive in some neuroendocrine tumors of different origins as well as in colorectal, gastric, endometrial, endocervical, ovarian, prostatic, renal, and mesothelial tumors [3-6].

Regarding primary breast cancer, some cases with TTF-1 positivity have been reported [7-11] (Table 1). We recently documented a case of breast cancer with positive expression of TTF-1 [9], and we subsequently wanted to establish the frequency of TTF-1 expression in a population-based setting of breast cancer.

**Table 1:** Overview of studies reporting TTF-1 positive primary breast cancers

**Reference**	**TTF-1 positiv/total**	**Study population**	**TTF-1 clone**
Ersahin et al.,	1/1	Case study	8G7G3/1
Int J SurgPathol 2009			
Christie et al.,	1/1	Case study	Unknown
IntJ Clin Exp Pathol 2010			
Klingen et al.,	1/1	Case study	SPT24
DiagnPathol 2010			
Robens et al.,	13/546	Unselected	SPT24
Am J SurgPathol 2010			
Sakuray et al.,	4/134	Unselected	8G7G3/1
Histopathol 2011			
Present study	7/247	Population-based	SPT24

## Materials and methods

We carried out immunostaining for TTF-1 on paraffin sections of 247 primary breast cancers selected from the population-based Norwegian Breast Cancer Screening Program. Vestfold County (Eastern Norway, 5% of the Norwegian population with around 230,000 inhabitants) was included in the Norwegian Breast Cancer Screening Program in 2004. A total of 37,977 women participated during the study period from 2004 to 2008; attendance rate was 71% and 76% during the first two screening rounds. During the first two rounds, 204 invasive screen-detected cancers were reported, and 46 invasive interval breast cancers following the prevalent and subsequent round were found. Three cases were excluded from this series: one screen-detected cancer had no residual tumor tissue for further investigation; one screen-detected tumor was diagnosed as a malignant phyllodes tumor, and one patient with interval cancer suffered from multiple metastases at the time of diagnosis, and no biopsy or surgery was therefore performed. Thus, a total of 247 invasive carcinomas were available for this population-based study, 202 screen-detected and 45 interval cancers.

One representative paraffin embedded block from each surgical specimen, including tumor tissue with adjacent peritumoral tissue, was selected for immunohistochemical staining with TTF-1. In 8 cases, preoperative tumour biopsies were selected for investigation because tumor tissue in surgical specimens was too scanty after neoadjuvant treatment for locally advanced breast cancers (6 cases) or routine pathology investigation (2 cases).

Immunohistochemical analyses were performed by routine methods on 4-5 μm-thick, formalin fixed, paraffin-embedded tissue sections. Briefly, after the slides were dried for 20-60 minutes in a 60°C oven, they were placed on the Ventana Benchmark automated immunostainer (Ventana Medical Systems, Tucson, AZ) and dewaxed. Heat-induced epitope retrieval was performed with Ventana`s CC1 retrieval solution for 30 minutes at 95°C to 100°C. Primary antibody Thyroid transcription factor-1 (TTF-1), clone SPT24 (Novocastra/Leica) at a 1:100 dilution, mouse monoclonal antibody, was applied to the sections at 37°C for 32 minutes. Presence of the antigen was visualized by using the Ultra View DAB detection kit (Ventana).

TTF-1 positive cases were evaluated for intensity of nuclear expression and expression area. A staining index (values 0-9), obtained as a product of staining intensity (0-3) and proportion of immunopositive tumor cells (≤10% = 1, 10-50% = 2, >50% = 3), was calculated as previously published [12]. Staining index ≥6 was considered to represent strong TTF-1 expression and a staining index <6 was considered a weak TTF-1 expression. Staining was recorded independently by three observers (TK, YC and PS). In cases recorded with different observer evaluation, consensus was obtained after discussion between all three observers. TTF-1 positive cases were also evaluated by Anti-Thyroglobulin staining (clone 2H11 + 6E1, Roche).

The cases were divided into two groups according to tumor size ≤ 2 cm or > 2 cm. Histological grade was recorded according to the Nottingham criteria [13]. Nodal status was reported as negative for lymph nodes without tumor or positive for one or more lymph nodes with tumor spread. Tumor cells invading lymphatic or blood vessels were examined on sections using D2-40 and CD31 as previously described [14]. Briefly, lymphatic vessel invasion (LVI) was recorded if tumor tissue was located within more than one D2-40 positive vessel structure, and blood vessel invasion (BVI) was reported when tumor cells were detected in one or more CD31 positive and D2-40 negative vessels. Methods and evaluation for Cytokeratin 5/6, P-cadherin, Ki67, estrogen receptor (ER), progesterone receptor (PR) and HER2 have been described previously [15].

Data were analyzed using the Fisher`s exact test (2×2 contingency table). P-values of 0,05 or less were considered significant.

The study was approved by the Regional Committee for Medical Research Ethics and protocols followed the guidelines by the Helsinki Declaration.

## Results

Among 247 primary breast cancer specimens in this study, 201 (81%) were invasive ductal carcinomas, 26 (11%) were invasive lobular carcinomas and the remaining 20 cases (8%) consisted of a variety of other types of breast carcinomas. 73 cases (30%) were grade 1, 122 (49%) grade 2 and 52 (21%) grade 3. We found 189 cases (77%) ≤2 cm and 58 cases (23%) > 2 cm. The median tumor diameter was 14 mm in the screening group and 15 mm in the interval group. 77 cases (31%) had metastases to one or more lymph nodes. 218 cases (88%) were estrogen receptor (ER) positive (by 10% cut-off value of stained cells); 157 cases (64%) were progesterone receptor positive. HER2 was score 3+ in 21 (8.5%) of the cases; score 2+ was observed in 20 cases (8%), including 2 with ISH amplification. In total, 23 cases were considered HER2 positive (9,3%), representing 15 (7,4%) of the screen-detected cancers and 8 (17,8%) of the interval tumors.

**Figure 1:**
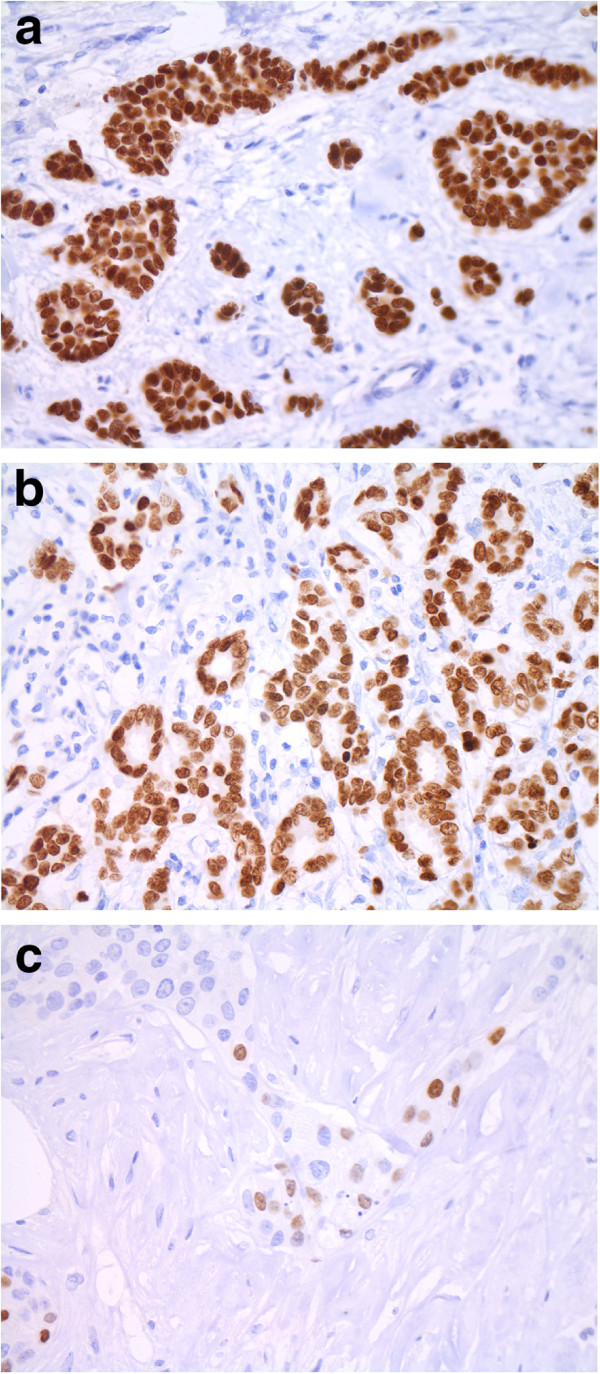
Breast carcinomas with strong (a, b) or weak (c) nuclear expression of TTF1 (x400).

TTF-1 (clone SPT24) expression was seen in 7 of 247 cases (2,8%) in this material (Table 2). Positive staining was shown in 6 surgical specimens and in one pre-operative core needle biopsy (Figure 1). Three cases had a high TTF-1 expression with a staining index of 9 (2 cases), and 6 (1 case), while 4 cases had a low TTF-1 expression with a staining index of 3 (1 case) and 2 (3 cases). There were 6 surgical specimens with carcinoma in situ in the periphery of invasive tumors, and 3 of these cases (#2, #4, #6) had TTF-1 expression in the carcinoma in situ component. Among the positive cases were six invasive ductal carcinomas and one case of invasive lobular carcinoma (#6). Follow-up time after tumor diagnosis for TTF-1 positive cases varied from 29-61 months, and one patient (#1) died from breast cancer. Mean distance from tumors to skin was 27 mm for TTF-1 positive cases. All 7 TTF-1 positive cases were negative for Anti-Thyroglobulin.

**Table 2:** Clinico-pathological and radiological characteristics of patients with TTF-1 positive primary breast cancers

**Patient number**	**Sample type**	**TTF-1 expression (staining index)**	**Tumor diameter (mm)**	**Grade**	**Tumor-positive lymph-nodes**	**ER**	**PR**	**Her2**	**CK 5/6 and/or P-cadherin**	**Ki 67 (%)**	**Mode of presentation**	**Mammo-graphy findings**	**Distance from skin (mm)**	**Follow-up time after tumor diagnosis (months)**	**Status**
1	SS	Weak (2)	27	3	1/12	-	-	+	+	50,5	Screening	Stellate opacity	47	29	DEAD
2	SS	Strong (9)	15	1	0/3	+	+	-	-	25,4	Interval	Irregular microlobulated density	36	61	AW
3	SS	Weak (3)	22	3	1/21	-	-	-	+	41,2	Interval	Diffuse density	30	38	AW
4	SS	Weak (2)	9	2	1/11	+	+	-	-	14,5	Screening	Irregular partially rounded mass	8	60	AW
5	SS	Weak (2)	21	3	1/14	-	-	-	+	41,2	Screening	Irregular density	18	49	AW
6	SS	Strong (9)	17	2	1/8	+	+	-	-	22,9	Screening	Irregular density	33	54	AW
7	CNB	Strong (6)	70	3	0/1	+	-	-	+	55,4	Interval	Lobulated mass density	16	60	AW

*SS*, Surgical specimen; *CNB*, Core needle biopsy; *ER*, Estrogen receptor; *PR*, Progesterone receptor; *DEAD*, Dead of disease; *AW*, Alive and well.

TTF-1 positivity was significantly more frequent with ER negative cases (14% vs. 1,4%; p = 0,004), Ki-67 staining by upper quartile (8,8% vs. 1,1%; p = 0,008), with CK 5/6 or P-cadherin positive cases (11,1% vs. 1,4%; p = 0,01), with lymph node positive cancers (5,2% vs. 1,8%; p = 0,03), with histological grade 3 tumors compared with grade 1 or 2 tumors (7,7% vs. 1,5%; p = 0,04), and with BVI positive cases (9,7% vs. 1,9%; p = 0,04).

TTF-1 positive tumors tended to be more frequent (although not significant) in interval cancers compared with screening-detected cancers (6,7% vs. 2,0%; P = 0,12), in PR negative tumors compared with PR positive tumors (4,4% vs. 1,9%; p = 0,26), in HER2 positive tumors compared to HER2 negative (4,8% vs. 2,7%; P = 0,47) and in LVI positive cancers (4,8% vs. 2,2%; p = 0,37) compared to LVI negative tumors.

## Discussion

In this population-based study, TTF-1 expression was found in 2,8% of the cases, almost the same frequency (2,4%) as Robens et al. [7]. Both studies used the SPT24 clone, but in the study by Robens a different autostainer (DAKO) was used on an unselected material with more biopsies than surgical specimens. Sakuray et al. [8] found 4/134 (3,0%) cases with positivity for TTF-1 (clone 8G7G3/1) in a study using tissue microarrays. All these three studies demonstrated similar frequencies of TTF-1 positivity (2-3%) despite some differences in clones and technical conditions.

There are two commercially available clones for monoclonal TTF-1 antibodies in immunohistological use; SPT24 and 8G7G3/1. SPT24 has been suggested to be more specific but less sensitive than 8G7G3/1 [16]. Reviewing previous literature indicates that TTF-1 positive breast cancer has been demonstrated with both clones (Table 1). Matoso et al. [17] found the same sensitivity for SPT24 and 8G7G3/1 in non-pulmonary tumors, under the same technical conditions for antigen retrieval and detection system. However, the SPT24 clone has a stronger nuclear staining and less non-specific cytoplasmic staining than 8G7G3/1 [18]. This clone is recommended by the Nordic Immunohistochemical Quality Control (NordiQC) [19].

Both Robens et al. and our present study demonstrated a significantly higher frequency of TTF-1 positivity in histological grade 3 tumors. As novel observations, we here find significantly more TTF-1 positive tumors with increased tumor cell proliferation (Ki67), a basal like phenotype (CK5/6 and/or P-cadherin expression), lymph node metastasis, ER negativity, and blood vessel invasion. These findings suggest an association between adverse prognostic factors and TTF-1 positivity in a small subgroup of breast cancers.

**Figure 2:**
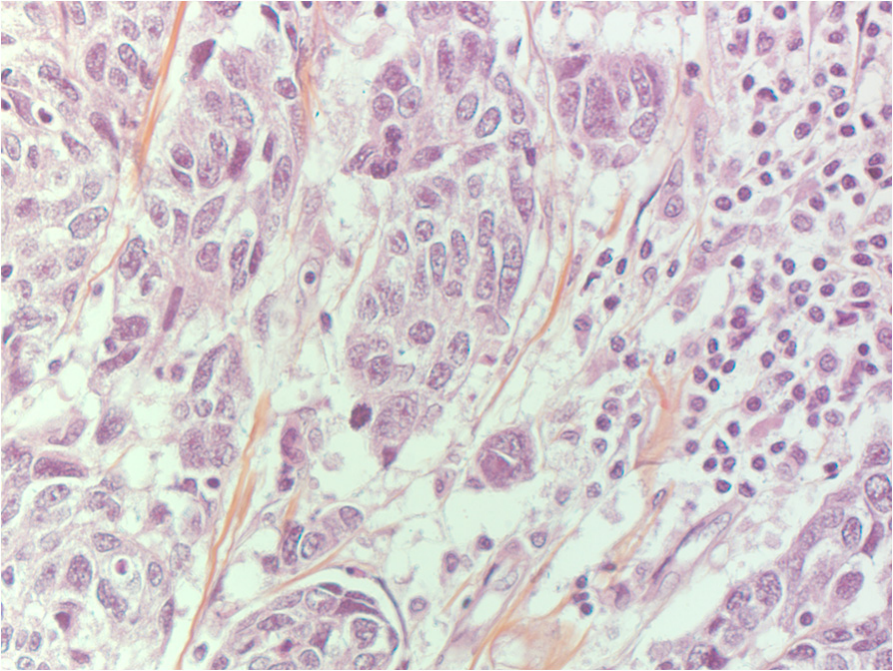
Basal-like morphology with marked nuclear pleomorphism and lymphocytic infiltration in one TTF-1 positive breast carcinoma.

In 2000, Perou and co-workers indentified five molecular subgroups of breast cancers (luminal A, luminal B, normal breast-like, Her2 positive or basal-like) by use of cDNA microarray and un-supervised cluster analysis [20], and several studies have since addressed the immunohistochemical surrogates to these subgroups [21]. The basal-like subgroup has variable expressions of high molecular-weight “basal” cytokeratins like CK5/6, CK14, CK17, myoepithelial markers (smooth muscle actin and p63), CD117, P-cadherin and EGFR [22]. Single markers like CK5/6 and P-cadherin have been used previously [23] and in the present study to define this subgroup. Basal-like tumors are often hormone receptor low or negative and Her2 negative (triple negative). Others have used more strict criteria with a “core basal profile”: ER negative, Her2 negative and CK5/6 and/or EGFR positive to define these tumors [24]. Morphologically, basal-like breast carcinomas are characterized by high histological grade, increased mitotic counts, marked nuclear pleomorphism, the presence of necrotic areas, pushing borders, and often marked lymphocytic infiltration [21]. A morphology with such basal-like features is demonstrated in one of our TTF-1 positive cases (Figure 2).

Secondary tumors in the breast are uncommon, with a reported frequency of 0,4-2% [25-27]. Studies [25,26,28] have previously shown that pulmonary carcinoma, malignant melanoma and lymphoma are the three most common secondary tumors. It could be argued that some of our TTF-1 positive cases represent metastases from lung cancer, but we regard this possibility as unlikely. First, six of our cases have carcinoma in situ in the periphery of the invasive tumor (except case #7). Second, in none of the patients was the tumor close to the skin, as is typical for metastases [19]. One patient (case #5) had a partially rounded mass located 8mm from the skin; however, metastasis was unlikely because of the presence of ductal carcinoma in situ and the fact that the patient survived more than 5 years after diagnosis. Third, one of the cases (case #6) showed typical features of lobular carcinoma, which argues against pulmonary origin.

It can be challenging to differentiate a primary breast carcinoma from a cancer metastasis. Pulmonary adenocarcinomas often demonstrate the same cytokeratin-profile as breast carcinomas with CK7 positivity and CK20 negativity. Also, pulmonary carcinomas can occasionally stain positive for ER, PR, mammaglobin or gross cystic disease protein [7,29-32]. Napsin A is a sensitive marker for lung adenocarcinoma [33], and a combination of TTF-1 and Napsin A may possibly be better than TTF-1 alone in confirming or excluding lung metastases to the breast. It is important that several markers are used in combination as a profile to differentiate between breast carcinomas and a metastatic lesion. Clinical and radiological information is also important in achieving the correct diagnosis. Information about known pulmonary tumors should always be included on the pathology request form as well as radiological information pertaining to breast lesions, such as morphology of the tumor and its distance from the skin, which can all be of valuable help in the diagnosis.

In conclusion, our population-based study indicates that TTF-1 expression may be present in 3% of primary breast cancers. In addition to previous observations, our novel findings indicate that TTF-1 expression is strongly associated with features of aggressive breast cancer and adverse prognostic factors, such as estrogen negativity, increased tumor cell proliferation, a basal-like phenotype, blood vessel invasion and lymph node positivity.

## Competing interests

The authors declare that they have no competing interests.

## Authors’ contributions

TK wrote the manuscript and participated in the histological evaluation. YC and PS participated in the histological evaluation and writing. IS participated in statistical analysis and writing. MG supplied the relevant radiological details and participated in writing. LAA participated in histological evaluation and writing of the manuscript. All authors read and approved the final manuscript.

## References

[B1] Berghmans T Paesmans M Mascaux C Martin B Meert AP Haller A Thyroid transcription factor 1–a new prognostic factor in lung cancer: a meta-analysis Ann Oncol 2006 17 11 1673 1676 10.1093/annonc/mdl287 16980598

[B2] Ordonez NG Thyroid transcription factor-1 is a marker of lung and thyroid carcinomas Adv Anat Pathol 2000 7 2 123 127 10.1097/00125480-200007020-00007 10721419

[B3] Wong NA Kamel H Sheffield EA Sohail M Positive immunostaining for thyroid transcription factor-1 in colorectal adenocarcinoma using the 8G7G3/1 monoclonal antibody J Clin Pathol 2008 61 9 1070 1071 10.1136/jcp.2008.058552 18755730

[B4] Kubba LA McCluggage WG Liu J Malpica A Euscher ED Silva EG Thyroid transcription factor-1 expression in ovarian epithelial neoplasms Mod Pathol 2008 21 4 485 490 10.1038/modpathol.2008.4 18246044

[B5] Siami K McCluggage WG Ordonez NG Euscher ED Malpica A Sneige N Thyroid transcription factor-1 expression in endometrial and endocervical adenocarcinomas Am J Surg Pathol 2007 31 11 1759 1763 10.1097/PAS.0b013e3181131e21 18059234

[B6] Bejarano PA Baughman RP Biddinger PW Miller MA Fenoglio-Preiser C al-Kafaji B, et al. Surfactant proteins and thyroid transcription factor-1 in pulmonary and breast carcinomas Mod Pathol 1996 9 4 445 452 8729987

[B7] Robens J Goldstein L Gown AM Schnitt SJ Thyroid transcription factor-1 expression in breast carcinomas Am J Surg Pathol 2010 34 12 1881 1885 10.1097/PAS.0b013e3181f884e8 21107096

[B8] Sakurai A Sakai Y Yatabe Y Thyroid transcription factor-1 expression in rare cases of mammary ductal carcinoma Histopathology 2011 59 1 145 148 10.1111/j.1365-2559.2011.03869.x 21771032

[B9] Klingen TA Chen Y Gundersen MD Aas H Westre B Sauer T Thyroid transcription factor-1 positive primary breast cancer: a case report with review of the literature Diagn Pathol 2010 5 37 10.1186/1746-1596-5-37 20565809 PMC2896353

[B10] Ersahin C Bandyopadhyay S Bhargava R Thyroid transcription factor-1 and “basal marker”–expressing small cell carcinoma of the breast Int J Surg Pathol 2009 17 5 368 372 10.1177/1066896909340275 19578049

[B11] Christie M Chin-Lenn L Watts MM Tsui AE Buchanan MR Primary small cell carcinoma of the breast with TTF-1 and neuroendocrine marker expressing carcinoma in situ Int J Clin Exp Pathol 2010 3 6 629 633 20661411 PMC2907125

[B12] Straume O Akslen LA Alterations and prognostic significance of p16 and p53 protein expression in subgroups of cutaneous melanoma Int J Cancer 1997 74 5 535 539 10.1002/(SICI)1097-0215(19971021)74:5<535::AID-IJC10>3.0.CO;2-5 9355977

[B13] Elston CW Ellis IO Pathological prognostic factors in breast cancer. I. The value of histological grade in breast cancer: experience from a large study with long-term follow-up. C. W. Elston & I. O. Ellis Histopathology 1991 19 403 410 Histopathology 2002 Sep;41(3A):151-2, discussion 10.1111/j.1365-2559.1991.tb00229.x 1757079

[B14] Mannelqvist M Stefansson I Salvesen HB Akslen LA Importance of tumour cell invasion in blood and lymphatic vasculature among patients with endometrial carcinoma Histopathology 2009 54 2 174 183 10.1111/j.1365-2559.2008.03201.x 19207942

[B15] Collett K Stefansson IM Eide J Braaten A Wang H Eide GE A basal epithelial phenotype is more frequent in interval breast cancers compared with screen detected tumors Cancer Epidemiol Biomarkers Prev 2005 14 5 1108 1112 10.1158/1055-9965.EPI-04-0394 15894660

[B16] Bisceglia M Galliani C Rosai J TTF-1 expression in breast carcinoma-the chosen clone matters Am J Surg Pathol 2011 35 7 1087 1088 10.1097/PAS.0b013e31821c2d47 21677546

[B17] Matoso A Singh K Jacob R Greaves WO Tavares R Noble L Comparison of thyroid transcription factor-1 expression by 2 monoclonal antibodies in pulmonary and nonpulmonary primary tumors Appl Immunohistochem Mol Morphol 2010 18 2 142 149 10.1097/PAI.0b013e3181bdf4e7 19887917 PMC2828524

[B18] Comperat E Zhang F Perrotin C Molina T Magdeleinat P Marmey B Variable sensitivity and specificity of TTF-1 antibodies in lung metastatic adenocarcinoma of colorectal origin Mod Pathol 2005 18 10 1371 1376 10.1038/modpathol.3800422 15861215

[B19] Nordiqc 2012 http://www.nordiqc.org/Run-33-B12-G2/Assessment/assessment-33-TTF1.htm

[B20] Perou CM Sorlie T Eisen MB van de Rijn M Jeffrey SS Rees CA Molecular portraits of human breast tumours Nature 2000 406 6797 747 752 10.1038/35021093 10963602

[B21] Reis-Filho JS Tutt AN Triple negative tumours: a critical review Histopathology 2008 52 1 108 118 18171422 10.1111/j.1365-2559.2007.02889.x

[B22] Cakir A Gonul II Uluoglu O A comprehensive morphological study for basal-like breast carcinomas with comparison to nonbasal-like carcinomas Diagn Pathol 2012 7 145 10.1186/1746-1596-7-145 23082819 PMC3488514

[B23] Arnes JB Brunet JS Stefansson I Begin LR Wong N Chappuis PO Placental cadherin and the basal epithelial phenotype of BRCA1-related breast cancer Clin Cancer Res 2005 11 11 4003 4011 10.1158/1078-0432.CCR-04-2064 15930334

[B24] Nielsen TO Hsu FD Jensen K Cheang M Karaca G Hu Z Immunohistochemical and clinical characterization of the basal-like subtype of invasive breast carcinoma Clin Cancer Res 2004 10 16 5367 5374 10.1158/1078-0432.CCR-04-0220 15328174

[B25] Hajdu SI Urban JA Cancers metastatic to the breast Cancer 1972 29 6 1691 1696 10.1002/1097-0142(197206)29:6<1691::AID-CNCR2820290637>3.0.CO;2-4 4337956

[B26] Georgiannos SN Chin J Goode AW Sheaff M Secondary neoplasms of the breast: a survey of the 20th Century Cancer 2001 92 9 2259 2266 10.1002/1097-0142(20011101)92:9<2259::AID-CNCR1571>3.0.CO;2-O 11745279

[B27] Vergier B Trojani M de Mascarel I Coindre JM Le Treut A Metastases to the breast: differential diagnosis from primary breast carcinoma J Surg Oncol 1991 48 2 112 116 10.1002/jso.2930480208 1921396

[B28] Klingen TA Klaasen H Aas H Chen Y Akslen LA Secondary breast cancer: a 5-year population-based study with review of the literature APMIS 2009 117 10 762 767 10.1111/j.1600-0463.2009.02529.x 19775345

[B29] Ishibashi H Suzuki T Suzuki S Niikawa H Lu L Miki Y Progesterone receptor in non-small cell lung cancer–a potent prognostic factor and possible target for endocrine therapy Cancer Res 2005 65 14 6450 6458 10.1158/0008-5472.CAN-04-3087 16024650

[B30] Nose N Uramoto H Iwata T Hanagiri T Yasumoto K Expression of estrogen receptor beta predicts a clinical response and longer progression-free survival after treatment with EGFR-TKI for adenocarcinoma of the lung Lung Cancer 2011 71 3 350 355 10.1016/j.lungcan.2010.06.009 20615575

[B31] Takeda Y Tsuta K Shibuki Y Hoshino T Tochigi N Maeshima AM Analysis of expression patterns of breast cancer-specific markers (mammaglobin and gross cystic disease fluid protein 15) in lung and pleural tumors Arch Pathol Lab Med 2008 132 2 239 243 18251583 10.5858/2008-132-239-AOEPOB

[B32] Wang LJ Greaves WO Sabo E Noble L Tavares R Ng T GCDFP-15 positive and TTF-1 negative primary lung neoplasms: a tissue microarray study of 381 primary lung tumors Appl Immunohistochem Mol Morphol 2009 17 6 505 511 10.1097/PAI.0b013e3181a8e809 19620839

[B33] Ye J Findeis-Hosey JJ Yang Q McMahon LA Yao JL Li F Combination of napsin A and TTF-1 immunohistochemistry helps in differentiating primary lung adenocarcinoma from metastatic carcinoma in the lung Appl Immunohistochem Mol Morphol 2011 19 4 313 317 10.1097/PAI.0b013e318205b059 21464700

